# Endothelial Plasticity: Shifting Phenotypes through Force Feedback

**DOI:** 10.1155/2016/9762959

**Published:** 2016-01-24

**Authors:** Guido Krenning, Valerio G. Barauna, José E. Krieger, Martin C. Harmsen, Jan-Renier A. J. Moonen

**Affiliations:** ^1^Cardiovascular Regenerative Medicine Research Group (Cavarem), Department of Pathology & Medical Biology, University Medical Center Groningen, University of Groningen, Hanzeplein 1 (EA11), 9713GZ Groningen, Netherlands; ^2^Laboratory of Genetics and Molecular Cardiology (LIM13), Heart Institute (InCor), University of São Paulo, Avenida Dr. Eneas C. Aguiar 44, 05403-000 São Paulo, SP, Brazil; ^3^Department of Physiological Sciences, Federal University of Espírito Santo (UFES), Avenida Marechal Campos 1468-Maruípe, 29043-900 Vitoria, ES, Brazil; ^4^Center for Congenital Heart Diseases, Department of Pediatric Cardiology, Beatrix Children's Hospital, University Medical Center Groningen, University of Groningen, Hanzeplein 1 (CA40), 9713GZ Groningen, Netherlands

## Abstract

The endothelial lining of the vasculature is exposed to a large variety of biochemical and hemodynamic stimuli with different gradients throughout the vascular network. Adequate adaptation requires endothelial cells to be highly plastic, which is reflected by the remarkable heterogeneity of endothelial cells in tissues and organs. Hemodynamic forces such as fluid shear stress and cyclic strain are strong modulators of the endothelial phenotype and function. Although endothelial plasticity is essential during development and adult physiology, proatherogenic stimuli can induce adverse plasticity which contributes to disease. Endothelial-to-mesenchymal transition (EndMT), the hallmark of endothelial plasticity, was long thought to be restricted to embryonic development but has emerged as a pathologic process in a plethora of diseases. In this perspective we argue how shear stress and cyclic strain can modulate EndMT and discuss how this is reflected in atherosclerosis and pulmonary arterial hypertension.

## 1. Introduction

Mechanical forces, laminar shear stress (LSS), and cyclic strain (CS) are two major and well-established regulators of vascular development and adaptation (reviewed in [1–3]). Vasculogenesis, which marks the onset of embryonic vascularization, is driven by hypoxia and initiated by the differentiation of endothelial cells from mesodermal angioblasts [4]. Herein, hypoxia inducible factor 1 alpha (HIF1*α*) induces the expression of vascular endothelial growth factor (VEGFa) which results in endothelial differentiation and proliferation. The endothelial cells derived from the blood islands form a vascular network known as the primitive capillary plexus [5]. This plexus is highly uniform without a clear vascular hierarchy.

With the interconnection of the vascular plexus and major vessels a functional loop is formed and, as the heart begins to beat, the physical flow of fluid is introduced [6]. Arteriogenesis ensues the remodeling of the vessels to accommodate the physical forces associated with the increased pressures and flow. The pulsatile nature of the blood flow generated by the beating heart produces a complex interplay between two distinct mechanical forces, namely, fluid shear stress (FSS), the frictional force exerted by the flow of blood, and cyclic strain (CS), which results from the distensibility of the vessel wall caused by pulsations and increased hydrostatic pressures. Adaptation to these forces, known as angioadaptation, results in a hierarchical vascular tree composed of arteries, arterioles, capillaries, venules, and veins [7].

In the adult vasculature, hemodynamic forces play a major role in the maintenance of vessel homeostasis [8]. The most important intimation for a role of hemodynamic forces in vascular homeostasis is found in the focal nature of most vascular diseases. For example, despite the fact that most risk factors for atherosclerosis development and progression are present at the systemic level, this inflammatory arterial disease preferentially develops in regions which are characterized by disturbed hemodynamic flow, typically encountered at the outer walls of vascular bifurcations and at the inner wall of vascular curvatures [9, 10]. Indeed, disturbances of fluid shear stress levels encountered at these locations are predictors of plaque location [11], associate with the increased mass transport of low-density lipoproteins (LDL) which build up the plaques [12], and are major determinants of plaque transition from stable plaques to high-risk unstable plaques and plaque rupture [13].

In recent years, it has become apparent that the endothelium plays a pivotal role in the development and progression of cardiovascular diseases [14–16]. The endothelial lining of the vasculature is exposed to a large variety of stimuli which require the endothelium to be highly plastic [17], as is reflected by the remarkable heterogeneity of endothelial cells in tissues and organs. Although this endothelial plasticity is essential during homeostasis, during disease, pathological stimuli might induce adverse plasticity which can contribute to disease. Herein, endothelial-mesenchymal transition (EndMT) and its contribution to neointima formation [18–21] is a perfect example.

In this perspective, we focus on how hemodynamic forces, that is, shear stress and cyclic strain, are sensed by the endothelium, how these forces modulate EndMT, and how this is reflected in vascular disease.

## 2. Endothelial-to-Mesenchymal Transition: The Hallmark of Endothelial Plasticity

Endothelial-to-mesenchymal transition (EndMT) was originally described as an embryonic phenomenon involved in cardiac valve formation [22]. EndMT has mainly been studied* in vitro*, where it is characterized by the loss of cell-cell adhesions and changes in cell polarity, resulting in a spindle-shaped morphology. Endothelial cell markers such as VE-Cadherin and PECAM-1 are repressed, whilst the expression of mesenchymal cell markers such as *α*-smooth muscle actin (*α*-SMA) and calponin is enhanced [23–26]. Functionally, endothelial cells acquire myofibroblast-like characteristics with contractile function, enhanced migratory phenotype, and increased extracellular matrix production [24]. In EndMT, endothelial functions, such as antithrombogenicity and angiogenic sprouting capacity, are lost [23, 24].

**Figure 1:**
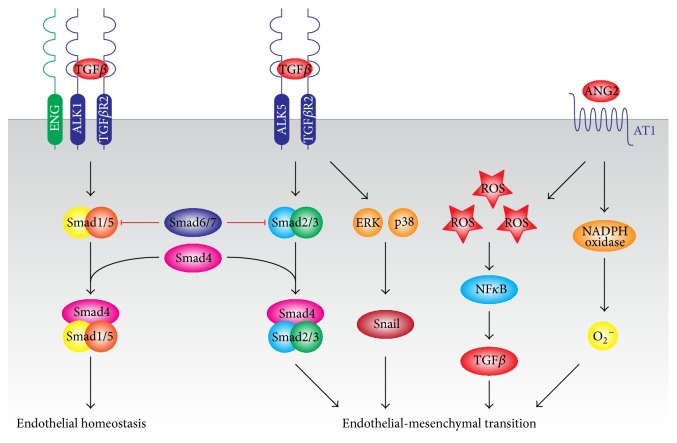
Mechanisms of endothelial-mesenchymal transition. EndMT is induced by TGF*β*-dependent and TGF*β*-independent mechanisms. Canonically, TGF*β*-induced activation of SMAD2/3 induces the expression of mesenchymal genes and repression of endothelial genes. Noncanonically, TGF*β*-induced activation of Erk1/2 and p38 MAPK activate the transcription factor Snail which induces mesenchymal differentiation and inhibits VE-Cadherin expression. Inflammatory signals and increased ROS production facilitate EndMT by increasing endogenous TGF*β* expression, in an NF*κ*B-dependent manner, creating a feed forward signaling mechanism. AT1 receptor can induce EndMT through activation of NADPH oxidase, resulting in increased ROS production and reduction of eNOS expression and activity.

Through extensive studies on EndMT* in vitro, *we have gained in-depth knowledge of the signaling cascades that regulate EndMT (Figure 1). Many signaling pathways are involved in the induction and progression of EndMT, wherein the transforming growth factor *β* (TGF*β*)/bone morphogenetic protein (BMP) superfamily plays a pivotal role.

In endothelial cells, canonical TGF*β* signaling occurs through the type II TGF*β* receptor (TGF*β*R2) which activates the type I TGF*β* receptor Activin-like kinase (ALK) 5, activating receptor-regulated Smad2/3, and leads to inhibition of cell proliferation and induction of EndMT [27–30]. In contrast, TGF*β* and bone morphogenetic proteins (BMP) can also bind the BMP type I receptor, ALK1, which activates receptor-regulated Smad1/5/8 and induces proliferation and inhibits EndMT [27–29]. The common-mediator Smad4 interacts with the receptor-regulated Smads and is required for signaling. The inhibitory Smads 6 and 7 block activation of the receptor-regulated Smads and thus inhibit TGF*β* signaling. The balance between ALK1 and ALK5 signaling pathways is partially regulated by an accessory type III TGF*β* receptor called endoglin, which stimulates ALK1-induced Smad1/5/8 responses and thus indirectly inhibits ALK5 signaling [29].

Besides canonical TGF*β* signaling, TGF*β* can induce EndMT noncanonically through the activation of the ERK1/2 and p38 MAPK signaling pathways and the downstream induction of the mesenchymal transcription factor Snail [31, 32] (Figure 1). Furthermore, Notch is known to promote TGF*β*-induced EndMT shifting the balance between different ALK1 and ALK5 signaling pathways in favor of ALK5 [33, 34].

Other signaling mechanisms can also induce EndMT, albeit often indirectly involving TGF*β* signaling. Proinflammatory molecules, such as IL1*β* and TNF*α*, synergize with TGF*β* in the induction of EndMT [35–37]. Besides, IL1*β* and TNF*α* induce the expression of Snail and Slug, two pivotal transcription factors in EndMT [38–40]. Reactive oxygen species (ROS) are potential stimulators of EndMT (Figure 1) by inducing endogenous TGF*β* expression and activating latent TGF*β* [41]. TGF*β* also stimulates ROS production, resulting in a positive feedback loop [41]. Besides, ROS activates NF*κ*B signaling which acts in synergy with TGF*β* in induction of EndMT [36, 42].

The AT1 receptor (angiotensin II receptor type I), a member of the G-protein coupled receptor family, mediates EndMT of human aortic endothelial cells* in vitro *[43] as well as in cardiac endothelial cells* in vivo* [44, 45]. Angiotensin II-induced EndMT can be mediated both by release of cytokines such as TGF*β* or through changes in the redox status of EC by the activation of NADPH oxidase system [45] which leads to higher superoxide production, NF*κ*B activation [42], and lower NO bioavailability through uncoupling eNOS [46, 47] (Figure 1).

In summary, TGF*β* signaling plays a dominant role as inducer of EndMT directly via both canonical and noncanonical signaling pathways. In recent years, it has become evident that EndMT is not restricted to embryogenesis but can also occur in adult life, where it contributes to organ and muscle fibrogenesis [48–52], cancer [53], and atherosclerosis [18–20]. In the following section, we argue how hemodynamic forces can modulate EndMT and how these mechanisms are reflected by vascular disease.

## 3. Hemodynamic Forces as Modulators of EndMT

### 3.1. Go with the Flow: Laminar Shear Stress Is a Hemodynamic Force Transduced by the Endothelium

Hemodynamic forces play a pivotal function in the maintenance of the vascular integrity. It is well established that high uniform laminar shear stress (LSS) has atheroprotective effects [54], which is evidenced by the occurrence of endothelial dysfunction [55], aneurysms [56], and atherosclerosis [57–59] at sites where LSS is reduced or worse, absent or disturbed.

A variety of signaling cascades are involved in endothelial mechanotransduction of LSS; however, identifying which pathways are primary or secondary to LSS sensing remains elusive as the initial mechanosensory complexes are not completely identified.

**Figure 2:**
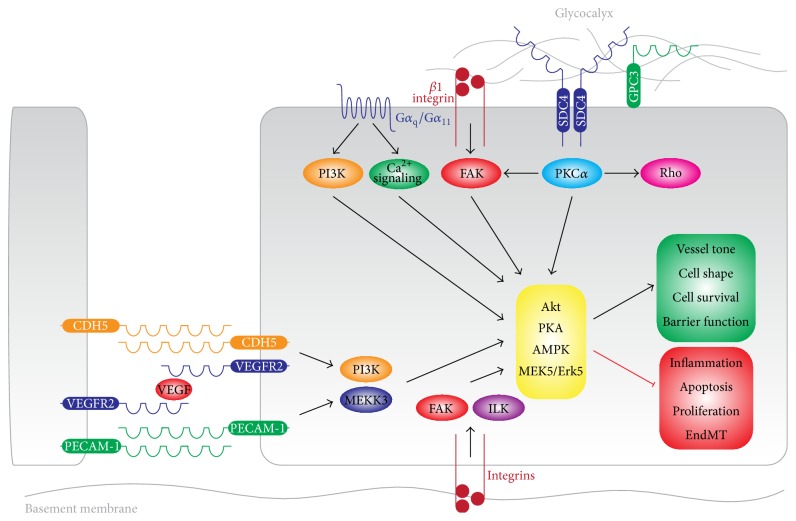
Endothelial shear stress sensing. Endothelial cells sense LSS through a number of mechanisms. Shear stress sensing through the endothelial glycocalyx is mediated by the syndecans and glypicans, which activate PKC signaling. Luminal *β*1-integrins also anchor to the endothelial glycocalyx and activate focal adhesion kinase (FAK). The G-protein-coupled receptors of G*α*_q_/G*α*_11_ sense shear stress and activate downstream PI3K and Ca^2+^ signaling. The junctional mechanosensory complex consisting of PECAM-1, VEGFR2, and VE-Cadherin mediates PI3K and MAPK (MEKK3) signaling. Signaling through these mechanosensors culminates in activation of Akt, PKA, AMPK, and MEK5/Erk5 which collectively maintain the endothelial phenotype.

Endothelial cells sense LSS through a number of mechanisms including the endothelial glycocalyx [60–62], growth factor receptors [63, 64], cell-cell adhesion molecules [65, 66], integrins [67], and G-protein-coupled receptors [68–70] (Figure 2). The endothelium is covered by a slimy layer of plasma proteins and glycoproteins, known as the glycocalyx, which is an organized hydrated mesh of negatively charged membranous macromolecules, proteoglycans, and glycosaminoglycans [71]. The proteoglycan core proteins are the membrane-bound glypican and the transmembrane syndecans. Syndecans directly associate with the cytoskeleton [72] and may thus directly transmit mechanical stresses to the nucleus or remote mechanotransducers [73, 74] (Figure 2). Indeed, disruption of the endothelial glycocalyx either genetically or by pharmacological inhibitors of glycocalyx producing enzymes renders the endothelium shear insensitive [60–62]. Although insights into glycocalyx signaling remain elusive, syndecans and glypicans are known to associate with the *β*1 integrins and mediate the activity of focal adhesion kinase (FAK) through protein kinase C-alpha (PKC*α*) [61, 62, 67, 73], resulting in the activation of Akt, Rho, and the endothelial nitric oxide synthase (eNOS). Combined, glycocalyx signaling enhances endothelial survival and maintenance of the endothelial barrier (Figure 2).

The protein complex consisting of PECAM-1, VEGFR2, and VE-Cadherin is a junctional mechanosensory complex that transduces hemodynamic forces into biochemical responses [75]. In this mechanosensory complex, VE-Cadherin functions as an initial adaptor which initiates the formation of the signaling complex, whereas PECAM-1 can directly transduce mechanical forces to the cytoskeleton [65]. VEGFR2, in association with VEGFa, initiates bidirectional activation of PI3K and MEKK3 which cause the downstream activation of Akt and MEK5/Erk5 signaling (Figure 2). Collectively, signaling through this junctional complex results in the activation of a number of transcription factors [63, 64] described below and loss of any one of its components ameliorates the endothelial response to LSS [2].

Additionally, the membrane bound G-protein-coupled receptors of type G*α*_q_/G*α*_11_ transmit mechanical forces in part through the activation of PI3K and intracellular calcium signaling [76–78]. Activation of G*α*_q_/G*α*_11_ proteins results in the activation of Akt and increased activation of eNOS (Figure 2).

Thus, the endothelium is highly sensitive to laminar shear stress and endothelial mechanosensing and transduction is achieved through a variety of cell-matrix and cell-cell receptor complexes. Endothelial mechanotransduction by these protein complexes culminates in the activation of the Akt, PKA, AMPK, and MEK5/Erk5 signaling cascades that ensure the maintenance of endothelial homeostasis and inhibit EndMT (Figure 2).

### 3.2. The Atheroprotective Effects of Laminar Shear Stress and the Inhibition of EndMT

It is well established that the extracellular signal-related kinase (Erk) 5, also known as MAPK7 and big-mitogen kinase-1 (BMK-1), has atheroprotective effects [54, 79, 80]. Erk5 is the only mitogen-activated protein kinase (MAPK) which is continuously activated by uniform LSS* in vitro *[18, 81] and several lines of evidence suggest that Erk5 transmits its atheroprotective effects through the activation of the transcription factors of the Krüppel-like factor (KLF) family [18, 82–84].

First, Krüppel-like factor 2 (KLF2) is an important shear stress-activated transcription factor, which exerts anti-inflammatory effects through inhibition of nuclear factor kappa B (NF*κ*B) activation [85] and anticoagulant effects by induction of thrombomodulin and repression of tissue factor and plasminogen activator inhibitor (PAI-1) [86]. Besides, KLF2 regulates antioxidative signaling [87], induces quiescence [88], and inhibits VEGFa-induced angiogenesis, barrier disruption, and cell proliferation [89].

Second, KLF2 inhibits the phosphorylation and nuclear translocation of Smad2 through induction of inhibitory Smad7 [90]. Moreover, KLF2 also suppresses the activator protein 1 (AP-1), an important cofactor for TGF*β*-dependent transcription [90]. Thus, KLF2 might directly inhibit EndMT by suppressing TGF*β* signaling.

The expression of KLF4, a close family member of KLF2, is similarly induced upon exposure to LSS [91, 92]. Significant conservation exists between KLF2 and KLF4 [82, 93]. In fact, almost 60% of MEK5 regulated genes are coregulated by KLF2 and KLF4 [93]. Similar to KLF2, KLF4 exerts anti-inflammatory effects through suppression of NF*κ*B activity [94]. KLF4-specific targets include forkhead box O1 (FOXO1) and vascular endothelial growth factor (VEGF) [93], both of which are known to inhibit TGF*β* signaling [48, 95].

Third, KLF4 plays an important role in cell-cycle regulation and differentiation. KLF4 increases the expression of several inhibitors of proliferation, while genes that promote proliferation are repressed [88, 96, 97]. As such, KLF4 might partially be responsible for the quiescence observed in endothelial cells exposed to high levels of uniform LSS.

Fourth, KLF4 is known to suppress mesenchymal differentiation through several mechanisms. KLF4 inhibits myocardin expression, a potent coactivator of serum response factor (SRF) [98, 99], and prevents SRF/myocardin from associating with mesenchymal gene promoters [100]. KLF4 can also directly bind the TGF*β* control element (TCE) in the promoter of mesenchymal genes, preventing transcription [101]. Furthermore, KLF4 interacts with Smad3, inhibiting its binding to Smad binding elements (SBE) and preventing transcription of mesenchymal genes [102]. Interestingly, KLF4 is also known to transcriptionally regulate VE-Cadherin [103]. Thus KLF4 might inhibit EndMT via multiple mechanisms.

Last, KLF2 and KLF4 can inhibit EndMT through the inhibition of ROS formation by eNOS-induced stimulation of superoxide dismutase (SOD), scavenging of ROS through NO production [104, 105], and facilitation of mitochondrial biogenesis [106].

In addition to the atheroprotective effects mediated by the KLFs, LSS induces the expression of inhibitor of DNA binding/inhibitor of differentiation proteins 1–3 (Id1–3) [107]. These dominant-negative regulators of basic helix-loop-helix DNA-binding transcriptional regulators play a role in lineage commitment, cell cycle control, and cell differentiation [108]. Id2 and Id3 repress Smad2-mediated gene transcription [109] and ectopic expression of Id2 and Id3 inhibits EMT [110]. Besides, Id3 is downregulated during EndMT [24] and Id1 has been shown to promote survival of endothelial cells and preserve the integrity of lung microvascular endothelial cells [111].

In summary, LSS plays a major role in endothelial homeostasis, wherein mechanical stimuli are transduced into biochemical signals which culminate in the expression of the KLF transcription factors. KLF2 and KLF4 govern endothelial thrombogenicity, inflammatory phenotype, permeability, proliferation, and redox state through a variety of mechanisms. In addition, the KLF transcription factors inhibit mesenchymal gene expression. Hence, the LSS-induced expression of the KLF transcription factors is pivotal in the inhibition of EndMT.

### 3.3. Disturbed Shear Stress: Inducer or Permitter of EndMT?

In contrast to the beneficial effects of LSS, oscillatory or nonuniform shear stress, further referred to as disturbed shear stress (DSS), has opposing effects on endothelial homeostasis; that is, DSS causes EC dysfunction. DSS is known to induce endothelial inflammatory activation [112–114]. DSS reduces the endothelial antithrombogenicity and increases the formation of reactive oxygen species (ROS), which further adds to the inflammatory activation and profibrotic signaling. During arteriogenesis, the screw-like curvatures of the collaterals convert LSS to DSS, which stimulates endothelial proliferation and MCP-1 activation, resulting in the recruitment of monocytes that are essential for remodeling of the vascular structure [115]. These mechanisms are reiterated during vascular pathology. Areas exposed to DSS are characterized by a high influx of leukocytes, which generates a microenvironment with excessive ROS production, high levels of TGF*β*, and inflammatory mediators such as IL1*β*, which all favor EndMT [36].

DSS induces ROS formation through several mechanisms. DSS leads to increased nicotinamide adenine dinucleotide phosphate (NADPH) oxidase (NOX) activity (Figure 4) which results in ROS production [116]. NADPH oxidase activity can induce xanthine oxidoreductase (XO), an important source of ROS [117]. Next, expression of NOX4, a subunit of vascular NADPH oxidase, is increased in response to DSS resulting in increased LDL oxidation [118]. Adding to this, intracellular glutathione, a powerful antioxidant, is greatly decreased by DSS [119].

DSS can also induce EndMT through direct activation of TGF*β* signaling (Figure 4). BMP4 is a potent inducer of EndMT [48], expression of which is inhibited by LSS [120]. In contrast, exposure to DSS induces BMP4 [121] expression in endothelial cells and contributes to ROS production and NF*κ*B activation [122]. Additionally, DSS induces TGF*β* expression and activation in an NF*κ*B-dependent manner [123].

Taken together, high LSS can inhibit EndMT via multiple mechanisms, either directly or indirectly interfering with TGF*β* signaling. DSS suppresses these protective mechanisms making endothelial cells more prone to microenvironmental cues which favor EndMT. Besides, DSS can induce EndMT directly through induction of BMP4 or via increased ROS production. Indeed, exposing aortic endothelial cells (which normally are exposed to LSS) to DSS by aortic banding efficiently induces EndMT* in vivo* in the absence of other stimuli [18]. Therefore, DSS acts both as a permitter and inducer of EndMT by either suppressing protective signaling or by directly inducing the transition process.

### 3.4. Cyclic Strain

Cyclic strain (CS) is defined as the repetitive mechanical deformation of the vessel during the cardiac cycle. Vascular CS can vary from 2 to 20%* in vivo* as a result of arterial wall expansion and contraction in response to pulsatile pressure changes [124]. CS plays an important role in the modulation of cell proliferation, migration, apoptosis, morphological changes, and alignment through the production of vasoactive substances such as nitric oxide (NO) [125], endothelin (ET-1), [126] and antioxidants [127, 128].

Physiologically, the level of CS is around 6–10% but this strongly varies throughout the vasculature. CS can increase with hypertension and artificial pulmonary ventilation (10% to 20%) and decrease with ageing due to vascular stiffening or acutely due to sepsis (2%–6%) [129]. CS levels up to 10% do not induce endothelial injury [130] but inhibit endothelial apoptosis through activation of PI3-kinase and Akt. In contrast, CS levels of over 15% induce apoptosis [131].

**Figure 3:**
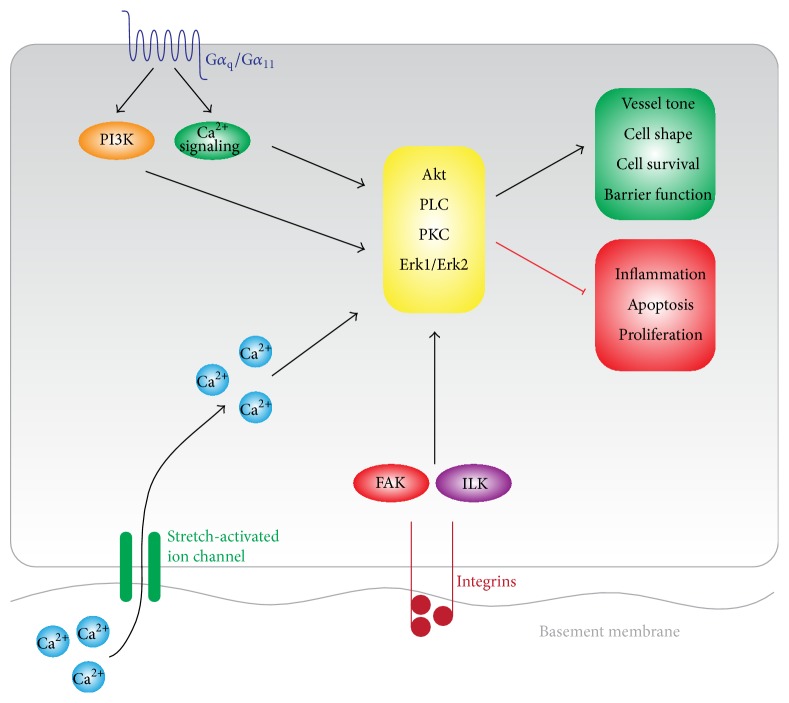
Endothelial cyclic strain sensing. Endothelial cells sense CS through a number of mechanisms. Stretch-sensitive ion channels undergo a conformational change during stretch which opens up the channel and induces Ca^2+^ flux from the extracellular space into the endothelial cell. Basal *β*1-integrins anchor to the endothelial basement membrane and activate focal adhesion kinase (FAK) and the integrin-linked kinases (ILK). The G-protein-coupled receptors of G*α*_q_/G*α*_11_ sense CS and activate downstream PI3K and Ca^2+^ signaling. Signaling through these stretch sensors culminates in activation of Akt, PKC, PLC, and Erk1/2 which collectively maintain the endothelial phenotype.

Endothelial cells sense CS through a number of mechanisms involving stretch-activated ion channels, integrins, and G-protein-coupled receptors. Stretch-activated ion channels play a pivotal role in CS sensing. Stretching of endothelial cells opens the ion channels allowing extracellular Ca^2+^ to enter the cell, thereby activating downstream phospholipase C (PLC) signaling [132] (Figure 3). Additionally, the cell-matrix adhesion families of integrins mediate CS signaling in endothelial cells by the activation of focal adhesion kinase (FAK) and integrin-linked kinase (ILK) complexes that result in the downstream activation of the small GTPase family Rho, PI3K, and Akt (Figure 3). Combined, these signals result in changes in endothelial morphology and the regulation of cell survival and cytokine production. However, to what extent CS induces these effects and the relation between the magnitude of CS and the endothelial integrin-mediated response remain elusive [133, 134]. The heterotrimeric G*α*_q_/*α*_11_ subunit of the G protein family is rapidly activated by CS and the intensity is related to the magnitude of the strain applied [135]. AT1 receptors bind and signal through all members of the G-protein family and are activated by mechanical forces* in vitro *and* in vivo*, independently of angiotensin II [136, 137].

**Figure 4:**
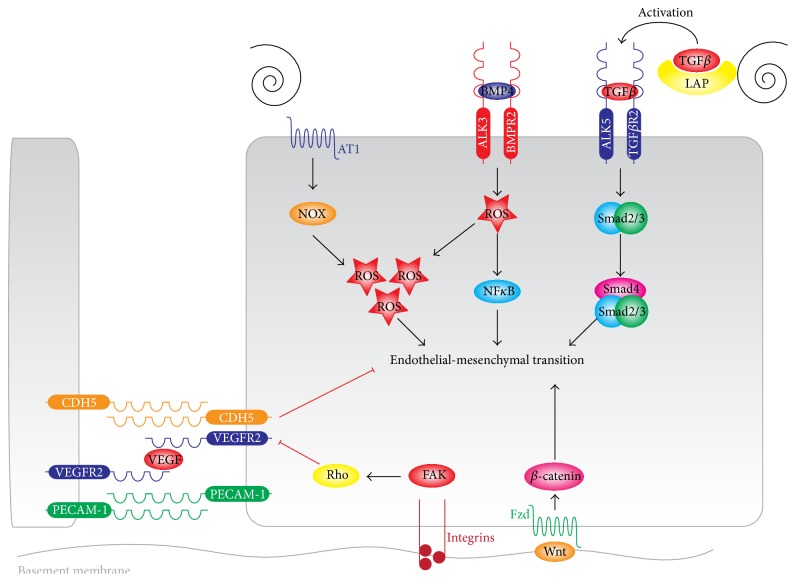
Disturbed shear stress and high cyclic strain signaling in endothelial-mesenchymal transition. Disturbed shear stress induces EndMT through several mechanisms. Disturbed shear stress activates latent TGF*β* by liberating it from LAP, after which TGF*β* can induce Smad2/3 signaling. Disturbed shear stress induces the expression of BMP4 which causes ROS formation and the activity of NF*κ*B. Lastly, disturbed shear stress induces NOX and XO activity resulting in the generation of ROS. All these signaling intermediates culminate in EndMT. Increased cyclic strain (>10%) induces the FAK-dependent activation of Rho-kinases. Rho activity causes the translocation of VE-Cadherin from the cell membrane into cytoplasmic vesicles and causes a reduction in endothelial cell-cell contacts. Cyclic strain-dependent Wnt-*β*-catenin activity induces EndMT in part by the induction of Snail and Slug and the further activation of Rho activity.

Stimulation of the CS sensors causes downstream activation of small GTPases that are essential for the biochemical transduction of the mechanical stimulus. The most extensively characterized members are Rho, Rac, and Cdc42, which have distinct effects on actin cytoskeleton, cell adhesion, and cell motility [138]. Rho kinase induces assembly of stress fibers and focal adhesions and Rac plays an important role in junction formation and integrity of the endothelial barrier [139]. The activity of the Rho pathway determines the direction and extent of stretch-induced stress fiber orientation in endothelial cells. This demonstrates on one hand that physically stressing a cell determines Rho activity (in a linear fashion) and that Rho activation directly correlates with physical shape shifting of cells through cytoskeletal reorganization [140, 141]. Also, preconditioning of endothelial cells at 18% CS enhanced the effects of prothrombotic stimuli to induce permeability of the endothelial monolayer, while 5% CS prevents thrombin-induced disruptive response and accelerates barrier recovery [142]. Corroboratively, CS in the 10–20% range causes activation of Rho and a reduction of basal Rac activity (Figure 4). In contrast, 5% stretch maintains the balance between Rho and Rac activity [143, 144]. These studies suggest a major role for amplitude-dependent CS in regulation of the endothelial barrier.

### 3.5. Cyclic Strain Sets the Stage for EndMT

Enhanced CS (>10%) potentiates EndMT of valvular endothelial cells in a manner dependent on both strain magnitude and direction [145]. Modest CS (10%) induces EndMT via amplified TGF*β*1 signaling, while high CS (20%) activates wnt/*β*-catenin signaling [145], a known inducer of EndMT in aortic endothelial cells [146, 147]. Both intensities decreased VEGFa signaling, a known inhibitor of EndMT [48]. Notably, mechanical stretch induces epithelial-to-mesenchymal transition (EMT) of renal tubular epithelial cells by induction of TGF*β*1 mRNA expression and activation of latent TGF*β* [146].

VE-Cadherin is a central component of endothelial adherens junctions. VE-Cadherin is a transmembrane glycoprotein that complexes via its cytoplasmic tail to *β*-catenin, which links VE-Cadherin to the cortical actin cytoskeleton [148]. The cellular localization of VE-Cadherin is dependent on the small GTPases Rho and Rac that when appropriately balanced stabilize VE-Cadherin at the cell membrane [149].

EndMT results in a loss of barrier function, that is, increased permeability, which correlates with loss of VE-Cadherin at the cell surface (Figure 4). In response to the increase of Rho activity by high levels of CS [141], VE-Cadherin is translocated from the membrane into the cytoplasm. The concomitant loss of complexed *β*-catenin and its nuclear translocation might therefore be a secondary mediator of EndMT. Indeed, Rho activity plays a pivotal role in TGF*β*1-induced EMT through induction of cytoskeleton remodeling and activation of the smooth muscle actin (SMA) promoter [150] and *β*-catenin/Rho signaling efficiently induces EndMT [32, 147, 151, 152]. This implies that supraphysiological CS is a direct inducer of EndMT (Figure 4).

Vascular remodeling is accompanied by changes in extracellular matrix (ECM) turnover, which result from alterations in the balance of matrix deposition and its proteolytic degradation. Matrix metalloproteinases (MMPs) represent the main group of proteases involved in remodeling of ECM [153]. An imbalance between the activity of MMPs and their tissue inhibitors (TIMPs) contributes to adverse remodeling. MMP2 induces matrix degradation during vascular remodeling [154] and TGF*β*2-induced EndMT is characterized by a marked increase in MMP2 [151]. Interestingly, biaxial CS induces endothelial MMP2 expression and regulates its secretion and activity [130, 155]. Additionally, in a model for intimal hyperplasia, stretched human saphenous vein grafts increased expression and activity of MMP2 throughout the vascular wall [156]. Taken together, cyclic strain induces MMP2 expression in endothelial cells thereby facilitating the migratory and proliferative phenotype acquired through EndMT.

Clearly, cyclic strain is not an ON/OFF switch of pathological vascular remodeling and EndMT, illustrated by the fact that endothelial cells react to CS in an amplitude-dependent manner. Stretching of endothelial cells may exert beneficial effects in physiological conditions (uniaxial, 5%–10%) but might induce advert effect during pathologies (strains <6% or >10%) such as hypertension. Reduced vascular distensibility is a common feature of vascular aging and is correlated with an increased risk of cardiovascular disease [157]. Loss of vessel wall compliance with age or increased CS during disease may blunt or aggravate the endothelial response to mechanical strain and induce EndMT. Whether and how this might contribute to pathophysiology remains to be determined.

### 3.6. Hemodynamic Forces Act in Concert

It should be emphasized that, for clarity reasons, LSS and CS were discussed as separate entities, yet they act in concert* in vivo*. For technical reasons, few studies have integrated both forces. However, fragmented evidence indicates that synchronicity between LSS and CS is important for vascular homeostasis, showing that asynchronicity between these hemodynamic forces induces a proatherogenic response through reduced expression of eNOS and cyclooxygenase-2 and increased expression of endothelin-1 and NF*κ*B [158–160]. These findings illustrate the importance of studying both entities in combination.

## 4. Hemodynamic Forces, EndMT, and Vascular Disease

EndMT contributes to vascular pathologies such as cardiac fibrosis [44, 50, 161], atherosclerosis [18, 20, 79], vascular restenosis [162], and the remodeling observed with pulmonary arterial hypertension [26]. As stated previously, the focal nature of these diseases, despite their systemic or genetic risk factors, intimates a pivotal role for hemodynamic forces in modulating these pathologies.

In atherosclerosis, levels of activated Smad2 are elevated especially at areas exposed to DSS [163]. Also, endothelial BMP4 expression is elevated at the site of atheroma formation and in calcifed atherosclerotic plaques, characterized by DSS [164], where also increased levels of ROS production are found [165], which all favor EndMT induction and progression. Indeed, at these sites, EndMT contributes to intimal hyperplasia and atherosclerosis development [18, 20].

In patients with pulmonary arterial hypertension (PAH), elevations in pulmonary venous pressure or long-term increases in blood flow such as those produced by intracardiac shunts result in increased hemodynamic loads and cause structural changes of the pulmonary vasculature. The remodeling response is characterized by endothelial dysfunction, intimal hyperplasia, muscularization of small peripheral vessels, and wall thickening in proximal vessels [166]. The increased hemodynamic load results in increased CS and DSS originating from flow reversal in the pulmonary trunk due to the curved path of the blood flow and dilatation of the pulmonary artery [167]. Pulmonary artery endothelial cells from patients with PAH display a hyperproliferative, apoptosis-resistant phenotype [168] concomitant with EndMT [24]. Interestingly, expression of the Early growth response protein 1 (Egr-1) is elevated in experimental flow-associated PAH [169, 170] and in the vessels of PAH patients with media hypertrophy and neointimal lesions, including plexiform lesions [169]. Egr-1 induces the expression of Snail and Slug, two important mediators of EndMT [171–173], which argues for a definite role of EndMT in the pathogenesis of PAH, as was recently evidenced in human PAH and in experimental models for PAH [19, 174, 175].

Besides, certain patients with familial PAH have mutations of the bone morphogenetic protein (BMP) receptor type II (BMPR2) gene or Activin-like kinase 1 (ALK1) gene [176, 177]. Under normal conditions, these receptors can stimulate Smad1/5/8 signaling in endothelial cells which inhibits EndMT [27, 28, 178]. Hence, impaired BMPR2 or ALK1 signaling renders the pulmonary endothelial cells from these patients more prone to EndMT. BMPR2 deficient rats show spontaneous pulmonary vascular remodeling with enhanced expression of Twist-1, an inducer of EndMT [175]. Corroboratively, adenoviral BMPR2 gene delivery to the pulmonary vascular endothelium in experimental models of PAH reduces the vascular remodeling [179].

## 5. Conclusion

In conclusion, hemodynamic forces clearly modulate vascular homeostasis and endothelial plasticity. Exposure of endothelial cells to proper amplitudes of LSS and CS safeguard the endothelial integrity through a variety of signaling cascades. Deviations in these hemodynamic forces, both by ageing or pathology, results in adverse endothelial plasticity and culminates in EndMT. Current advances on endothelial mechanotransduction can provide us with many new insights into the regulation of endothelial plasticity.
